# Evaluation of Interstitial Fluid Volume and Diffusivity in Patients With Idiopathic Normal Pressure Hydrocephalus Using Spectral Diffusion Analysis

**DOI:** 10.1002/jmri.29834

**Published:** 2025-07-04

**Authors:** Shota Ishida, Shigeki Yamada, Tatsuya Oki, Tomohiro Otani, Shinnosuke Hiratsuka, Shigeo Wada, Mitsuhito Mase, Yoshiyuki Watanabe

**Affiliations:** ^1^ Department of Radiological Technology, Faculty of Medical Science Kyoto University of Medical Science Nantan Japan; ^2^ Department of Radiology Shiga University of Medical Science Otsu Japan; ^3^ Department of Neurosurgery Nagoya City University Graduate School of Medical Science Nagoya Japan; ^4^ Interfaculty Initiative in Information Studies/Institute of Industrial Science The University of Tokyo Tokyo Japan; ^5^ Department of Mechanical Science and Bioengineering Graduate School of Engineering Science, The University of Osaka Toyonaka Japan

**Keywords:** diffusion imaging, glymphatic system, hakim disease, idiopathic normal pressure hydrocephalus, interstitial fluid, spectral analysis

## Abstract

**Background:**

Changes in interstitial fluid (ISF) dynamics are potential contributors to idiopathic normal pressure hydrocephalus (iNPH). Spectral diffusion analysis provides surrogate markers for ISF volume (*F*_int_) and diffusivity (*D*_int_).

**Purpose:**

To compare *F*_int_ and *D*_int_ between patients with iNPH and healthy controls (HCs).

**Study Type:**

Retrospective.

**Population:**

34 patients with iNPH (24 males, 10 females; 76.9 ± 7.6 years) and 132 HCs (46 males, 86 females; 47.5 ± 16.9 years; three age groups: < 40 years, 40–59 years, and ≥ 60 years).

**Field Strength/Sequence:**

3.0 T/Single‐shot spin‐echo echo‐planar imaging.

**Assessment:**

*F*_int_ and *D*_int_ were computed using non‐negative least squares and measured in eight regions, including the centrum semiovale (CSO), frontal white matter (FWM), and lenticular nucleus (LN). The regions of interest of iNPH were categorized as either periventricular hyperintensity (PVH) regions (iNPH_PVH_ group) or non‐PVH regions (iNPH group). *F*_int_ and *D*_int_ were compared among groups, and correlations with age were analyzed.

**Statistical Tests:**

Kruskal–Wallis test with Dunn's multiple comparison post hoc test. Spearman correlation coefficient. *p* < 0.05 was considered statistically significant.

**Results:**

In the CSO, iNPH_PVH_ group *F*_int_ values (40.3% ± 18.4%) exceeded those of other groups, while *D*_int_ values (1.53 ± 0.63 [× 10^−3^ mm^2^/s]) were significantly higher than those in younger HCs and the iNPH group. In the FWM, both *F*_int_ (53.0% ± 18.9%) and *D*_int_ (1.64 ± 0.50 [× 10^−3^ mm^2^/s]) were significantly higher in the iNPH_PVH_ group than in the other groups. In the LN, *F*_int_ correlated positively with age, regardless of the iNPH group's inclusion (without iNPH, *ρ* = 0.2114; with iNPH, *ρ* = 0.3044).

**Data Conclusion:**

Spectral diffusion analysis in iNPH demonstrated that ISF volume and diffusivity increased in PVH regions of the CSO and FWM, whereas ISF dynamics outside PVH regions may not differ significantly from those in HCs.

**Evidence Level:**

3.

**Technical Efficacy:**

Stage 1.

**Summary**

Plain language summary

○ Interstitial fluid (ISF) is the liquid found in the spaces between brain cells.
○ Changes in its behavior may contribute to idiopathic normal pressure hydrocephalus (iNPH), a treatable form of dementia.
○ We compared surrogate markers that indicate ISF behavior between patients with iNPH and healthy controls, and observed that these markers changed in specific areas of the brain where fluid from the brain's ventricles permeates into the brain tissue.
○ However, outside of these affected areas, ISF dynamics in iNPH may not differ significantly from those in healthy controls.
○ These findings help us better understand ISF dynamics in iNPH.

## 1 Introduction

Idiopathic normal pressure hydrocephalus (iNPH), also known as Hakim disease, is a treatable form of dementia characterized by gait disturbance, urinary incontinence, and cognitive impairment, managed with shunt implantation [1, 2, 3]. Cerebrospinal fluid (CSF) dynamics and intracranial biomechanical properties have been studied [4, 5, 6, 7, 8]. Additionally, the glymphatic system, including interstitial fluid (ISF) dynamics, has been explored to better understand brain waste clearance [9]. Notably, delayed gadolinium‐based contrast agent clearance from the brain parenchyma in iNPH suggests glymphatic hypofunction [10]. Furthermore, the loss of perivascular aquaporin‐4 may impair ISF dynamics in iNPH [11]. While aging and altered CSF and/or ISF dynamics are considered potential contributors to iNPH, its underlying pathophysiology remains largely unelucidated [1].

Diffusion magnetic resonance imaging (MRI) has been utilized to investigate intracranial fluid dynamics in iNPH, including intravoxel incoherent motion (IVIM) analysis and the examination of cardiac cycle‐associated temporal changes in the apparent diffusion coefficient [12, 13, 14]. Diffusion tensor imaging (DTI) analysis along the perivascular space (PVS) has been used to quantify glymphatic activity; however, its physiological interpretation remains debated [15, 16, 17, 18]. Another DTI study reported increased free water content in the periventricular white matter of iNPH [19].

Spectral diffusion analysis (SDA) is a variant of IVIM analysis [20, 21]. While conventional IVIM analyses derive two or three diffusion coefficients (DCs) and their fractions from intravoxel diffusion signal decay, SDA imposes no constraints on the number of diffusion compartments and computes a continuous diffusion spectrum. This spectrum is integrated into three components: the parenchymal diffusion, intermediate diffusion, and microvascular pseudo‐diffusion components, using predefined DC ranges. Previous studies have demonstrated that the fraction and DC of the intermediate diffusion component, denoted as *F*_int_ and *D*_int_, respectively, serve as surrogate markers for ISF volume and diffusivity [20, 21]. Furthermore, SDA offers shorter scan times than DTI and is less sensitive to overfitting compared with conventional IVIM analyses [20].

The hypothesis was that SDA could provide insight into ISF dynamics in iNPH. This study aimed to compare ISF volume and diffusivity between iNPH and healthy controls (HCs) and to evaluate their relationships with age.

## 2 Materials and Methods

### 2.1 Spectral Diffusion Analysis

**FIGURE 1:**
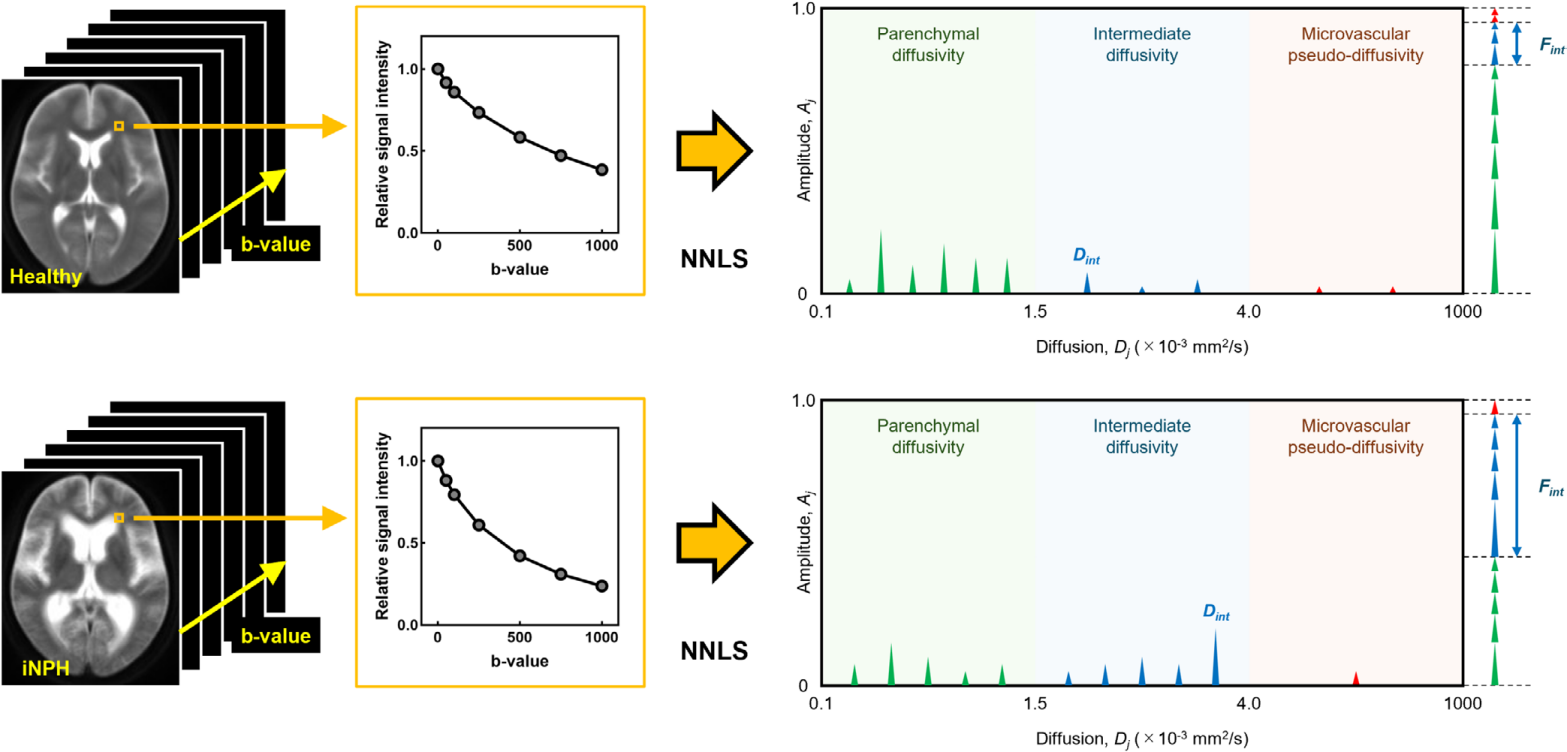
Conceptual diagram of spectral diffusion analysis depicting the diffusion coefficient *D*_*j*_ with its amplitude *A*_*j*_ and their differences between the white matter region in healthy controls (HCs; upper) and periventricular hyperintensity region in patients with idiopathic normal pressure hydrocephalus (iNPH; lower). In this example, the fractions of parenchymal diffusion, intermediate diffusion, and microvascular pseudo‐diffusion components for HCs are set at 0.80, 0.15, and 0.05, respectively, whereas for iNPH, they are set at 0.45, 0.50, and 0.05. NNLS, non‐negative least squares.

Figure 1 illustrates the conceptual framework of SDA. The diffusion signal decay is modeled as the sum of multiple mono‐exponential diffusion compartments, expressed as:

(1) $$\frac{S\left(b_{i}\right)}{S\left(b_{0}\right)}=\sum_{j=1}^{N}A_{j}\times \exp\left(-b_{i}D_{j}\right)$$

where *S*(*b*_*i*_) is the diffusion signal of the *i*th *b*‐value (*i* = 1, 2, …, *M*), and *S*(*b*_0_) is the diffusion signal at b‐value zero. *N* is the number of diffusion compartments, *A*_*j*_ is the amplitude of the *j*th diffusion compartment, and *D*_*j*_ is the *j*th DC, defined as a dictionary.

Equation 1 is solved by the non‐negative least squares (NNLS) algorithm for all *A*_*j*_ subject to *A*_*j*_ ≥ 0. Each component of the linear system for NNLS (i.e., *Ax* = *b*) is formulated as follows:

(2) $$\begin{array}{c} A=\left[\begin{array}{ccc} \exp\left(-b_{1}D_{1}\right) & \cdots & \exp\left(-b_{1}D_{N}\right)\\ \vdots & \ddots & \vdots \\ \exp\left(-b_{M}D_{1}\right) & \cdots & \exp\left(-b_{M}D_{N}\right) \end{array}\right] \end{array}$$

(3) $$\begin{array}{c} x=\left[\begin{array}{c} A_{1}\\ \vdots \\ A_{N} \end{array}\right] \end{array}$$

(4) $$\begin{array}{c} b=\left[\begin{array}{c} S\left(b_{1}\right)/S\left(b_{0}\right)\\ \vdots \\ S\left(b_{M}\right)/S\left(b_{0}\right) \end{array}\right] \end{array}$$

Amplitudes were integrated into three diffusion components based on the following ranges: the parenchymal diffusion (0.1 < *D* < 1.5 [× 10^−3^ mm^2^/s]); intermediate diffusion (1.5 ≤ *D* ≤ 4.0 [× 10^−3^ mm^2^/s]); and microvascular pseudo‐diffusion (4.0 < *D* < 1000 [× 10^−3^ mm^2^/s]) [20, 21]. The fraction of the intermediate diffusion component (*F*_int_) was computed by summing the amplitudes within its range [20, 21]. Similarly, the DC of this component (*D*_int_) was determined by selecting the dictionary‐defined DC value corresponding to the maximum amplitude position within this range [21].

### 2.2 Simulation Study

Prior to the in vivo study, Monte Carlo simulations were conducted using MATLAB 2024a (MathWorks, Natick, MA) to evaluate the effect of b‐value settings and compare two dictionaries by evaluating the accuracy of *F*_int_ and *D*_int_. This was necessary because of the retrospective nature of the study and because the current b‐value settings (0, 50, 100, 250, 500, and 1000 s/mm^2^) differ from those in previous studies, which used 15 b‐values: 0, 5, 7, 10, 15, 20, 30, 40, 50, 60, 100, 200, 400, 700, and 1000 s/mm^2^ [20, 21]. Previous studies employed a dictionary of 200 logarithmically spaced DCs ranging from 0.1 to 1000 [× 10^−3^ mm^2^/s], denoted as Log_200_ [20, 21]. Conversely, an alternative dictionary setting, Lin_200_, was implemented, containing 200 DCs with 66, 68, and 66 DCs linearly distributed across the ranges of parenchymal diffusion, intermediate diffusion, and microvascular pseudo‐diffusion components, respectively. The intermediate diffusion component includes boundary values, resulting in two additional points compared with the other components. Consequently, three conditions were compared: (1) the current *b*‐value setting with Lin_200_; (2) current *b*‐value setting with Log_200_; and (3) previous study setting (15 *b*‐values with Log_200_) [20, 21].

To synthesize simulation signals, the number of diffusion compartments (*N* in Equation 1) was defined to range from 1 to 50. For each *N*, 1000 patterns of DC combinations were sampled uniformly and randomly from the ground truth range: 0.1–1000 [×10^−3^ mm^2^/s]. If DCs were generated uniformly from this range, most would originate from the microvascular pseudo‐diffusion component. To prevent this bias, the algorithm was designed to generate DCs from each diffusion component with equal probability. Additionally, the amplitude of each diffusion compartment was randomly defined, ensuring that the sum of all amplitudes equaled 1. After generating theoretical diffusion signals using (Equation 1), Rician noise was incorporated by calculating magnitude signals after adding complex Gaussian noise sampled from independent and identical distributions to the theoretical signals [22]. The standard deviation (SD) range of the Gaussian distribution was set relative to signal intensity at *b* = 0 (*S*_0_) and defined as 0%–5% *S*_0_, generating 50 equally spaced SDs. Consequently, NNLS was applied to 2,500,000 diffusion signal decay patterns (50 diffusion compartments × 1000 DC combinations × 50 SDs). The accuracy of *F*_int_ and *D*_int_ was evaluated using the normalized root mean squared error (nRMSE).

(5) $$\begin{array}{c} \text{nRMSE}=\frac{1}{{\bar{x}}_{\mathrm{GT}}}\sqrt{\frac{1}{n}\sum_{i=1}^{n}{\left(x_{i}^{\mathrm{GT}}-x_{i}^{\mathrm{Est}}\right)}^{2}} \end{array}$$

where ${\bar{x}}_{\mathrm{GT}}$ is the mean of the ground truth, *n* denotes the number of data points, $x_{i}^{\mathrm{GT}}$ is the *i*th ground truth value, and $x_{i}^{\mathrm{Est}}$ is the *i*th estimated value.

### 2.3 In Vivo Study Population

This study retrospectively analyzed diffusion‐weighted images (DWIs) from a previous study that received institutional review board approval, followed a prospective observational design, and was conducted in accordance with the Declaration of Helsinki [7]. From November 2020 to February 2022, 133 HCs aged ≥ 20 years with no history of brain injury, brain tumor, or cerebrovascular disease on prior brain MRI, or who had never undergone brain MRI and had no neurological symptoms, including cognitive impairment, underwent MRI after providing informed consent. Although three HCs were incidentally found to have small (maximum diameter < 2 mm) unruptured intracranial aneurysms, they were included, as these findings were unlikely to affect ISF dynamics. One volunteer aged 84 years was excluded due to a history of head surgery more than 30 years prior. Consequently, 132 HCs were included and categorized into three age groups: under 40 years (*n* = 47, 16 males, 31 females; 29.3 ± 5.5 years), 40–59 years (*n* = 49, 15 males, 34 females; 49.1 ± 5.9 years), and ≥ 60 years (*n* = 36, 15 males, 21 females; 69.3 ± 6.8 years).

Patients' imaging data from the previous study were utilized in an opt‐out manner after anonymizing personal information in a linkable format [7]. A board‐certified neurosurgeon with expertise in imaging diagnosis of iNPH (S.Y., 28 years of experience) diagnosed the following findings. Among 44 patients suspected of having normal pressure hydrocephalus (NPH), five diagnosed with secondary NPH following subarachnoid hemorrhage, intracerebral hemorrhage, and severe meningitis, along with three diagnosed with congenital/developmental etiology NPH, were excluded [23, 24, 25, 26]. According to the Japanese guidelines for iNPH management, 36 patients were diagnosed with iNPH, exhibiting radiological findings of disproportionately enlarged subarachnoid space hydrocephalus, characterized by ventricular dilatation, enlarged Sylvian fissures, and narrow sulci at the high convexity, along with the clinical trial of gait disturbance, cognitive impairment, and/or urinary incontinence [27, 28]. Of these, two patients were excluded due to the unavailability of pre‐shunt DWI data. Consequently, 34 patients with iNPH (24 males, 10 females; 76.9 ± 7.6 years) were included.

### 2.4 In Vivo Data Acquisition and Image Processing

DWIs were acquired using a 3.0 T MRI (Discovery MR750W, GE HealthCare, Waukesha, WI). Single‐shot spin‐echo diffusion echo‐planar imaging was performed with the following parameters: repetition time, 6666 ms; echo time, 78 ms; slice thickness, 3.0 mm; field of view, 220 × 220 mm; acquisition matrix, 128 × 192; *b*‐values, 0, 50, 100, 250, 500, and 1000 s/mm^2^; and motion‐probing gradients, three orthogonal directions.

**FIGURE 2:**
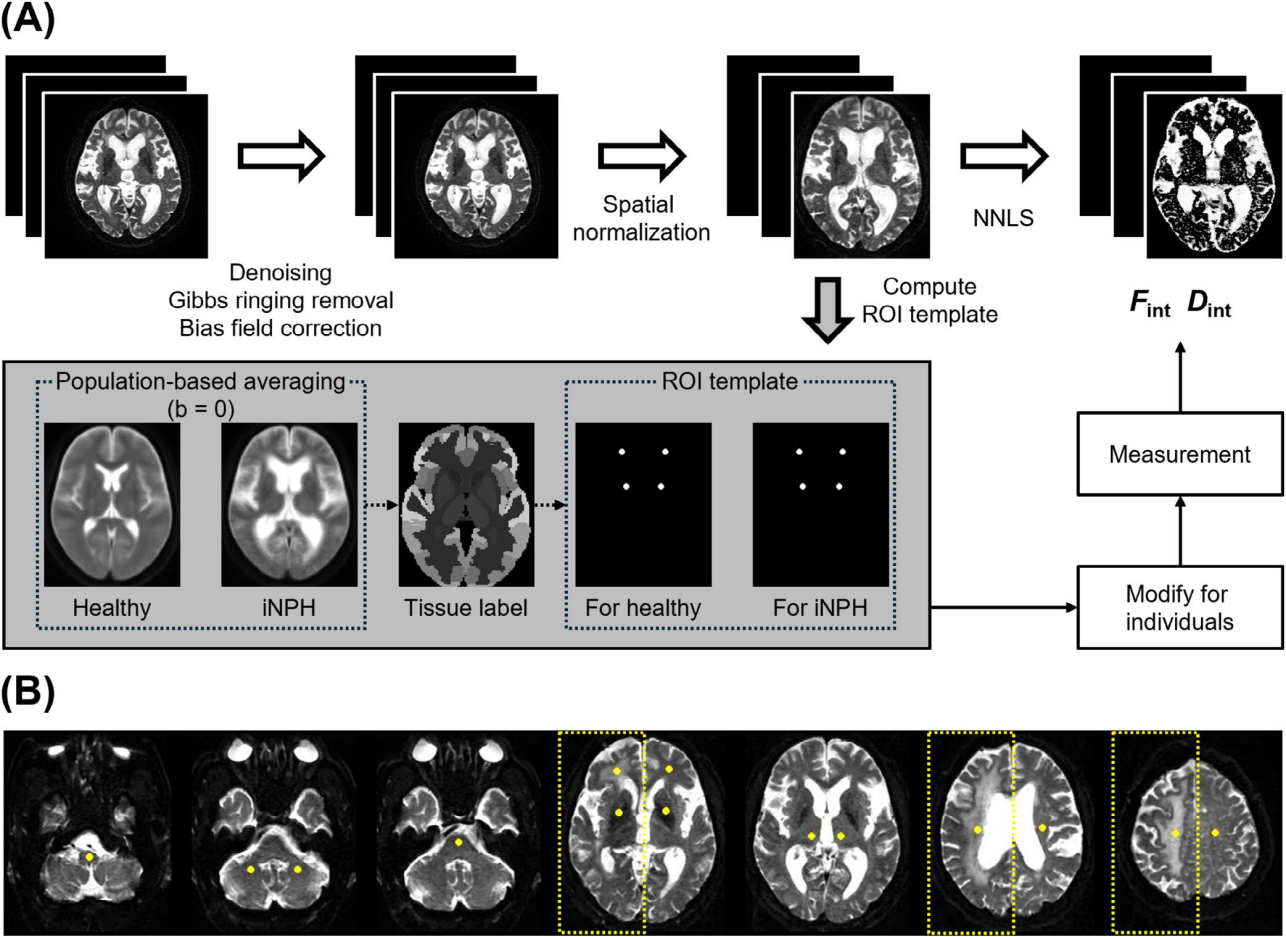
Data analysis procedures (A) and the locations of regions‐of‐interest (ROIs) in representative patients with idiopathic normal pressure hydrocephalus (iNPH; B). (A) The procedures included image pre‐processing, parameter estimation using non‐negative least squares (NNLS), and the creation of an ROI template. (B) Yellow dashed areas in the examples of ROI locations indicate another representative patient with iNPH, whose ROIs in the frontal white matter, corona radiata, and centrum semiovale were categorized into the iNPH periventricular hyperintensity (PVH; iNPH_PVH_) group.

Figure 2 illustrates the image processing workflow and regions of interest (ROI) locations. First, denoising and Gibbs ringing removal were performed using DIPY, followed by bias field correction using MRtrix3 [29, 30]. Motion correction was not performed as no significant motion artifact was identified. Subsequently, all DWIs were spatially normalized to the canonical template using SPM12. *F*_int_ and *D*_int_ maps were computed using NNLS and Lin_200_ (Figure 2A). For semi‐automatic measurement, an ROI template was created from the normalized b0 image and Neuromorphometrics tissue probability atlas (http://Neuromorphometrics.com/) obtained under an academic subscription and originating from the OASIS project (http://www.oasis‐brains.org/). The template included the following regions: the medulla oblongata, pons, bilateral cerebellar white matter, bilateral thalamus, bilateral lenticular nucleus (LN), bilateral frontal white matter (FWM), bilateral corona radiata, and bilateral centrum semiovale (CSO). Consequently, 14 circular ROIs with a seven‐voxel diameter were assigned to each participant (Figure 2B). When applying the ROI template, slight adjustments were made by a single observer (S.I., 11 years of experience in neuro MRI) without testing inter‐rater reliability to accommodate individual anatomical variations, as distortion correction was unavailable. These modifications also aimed to minimize contamination between white matter hyperintensity and isointensity regions. The ROIs of iNPH were classified into two groups through visual assessment and signal intensity thresholding: those located in periventricular hyperintensity (PVH) regions, categorized as the iNPH_PVH_ group, and the remaining ROIs, categorized as the iNPH group.

### 2.5 Statistical Analyses

GraphPad Prism 9.3 (GraphPad Software Inc., La Jolla, CA) was used. Since not all groups' data exhibited normality by the Shapiro–Wilk test, *F*_int_ and *D*_int_ values were compared using the Kruskal–Wallis test, followed by Dunn's multiple comparison post hoc test. The relationships between *F*_int_, *D*_int_, and age were assessed using the Spearman correlation coefficient. *p* < 0.05 was considered statistically significant. Notably, if the measured *F*_int_ values were zero, both *F*_int_ and *D*_int_ for that ROI were excluded from statistical analysis. Additionally, a receiver operating characteristic analysis was performed, and the results are presented in the Supporting Information (Figure S1 and Table S1).

## 3 Results

### 3.1 Simulation Study

**FIGURE 3:**
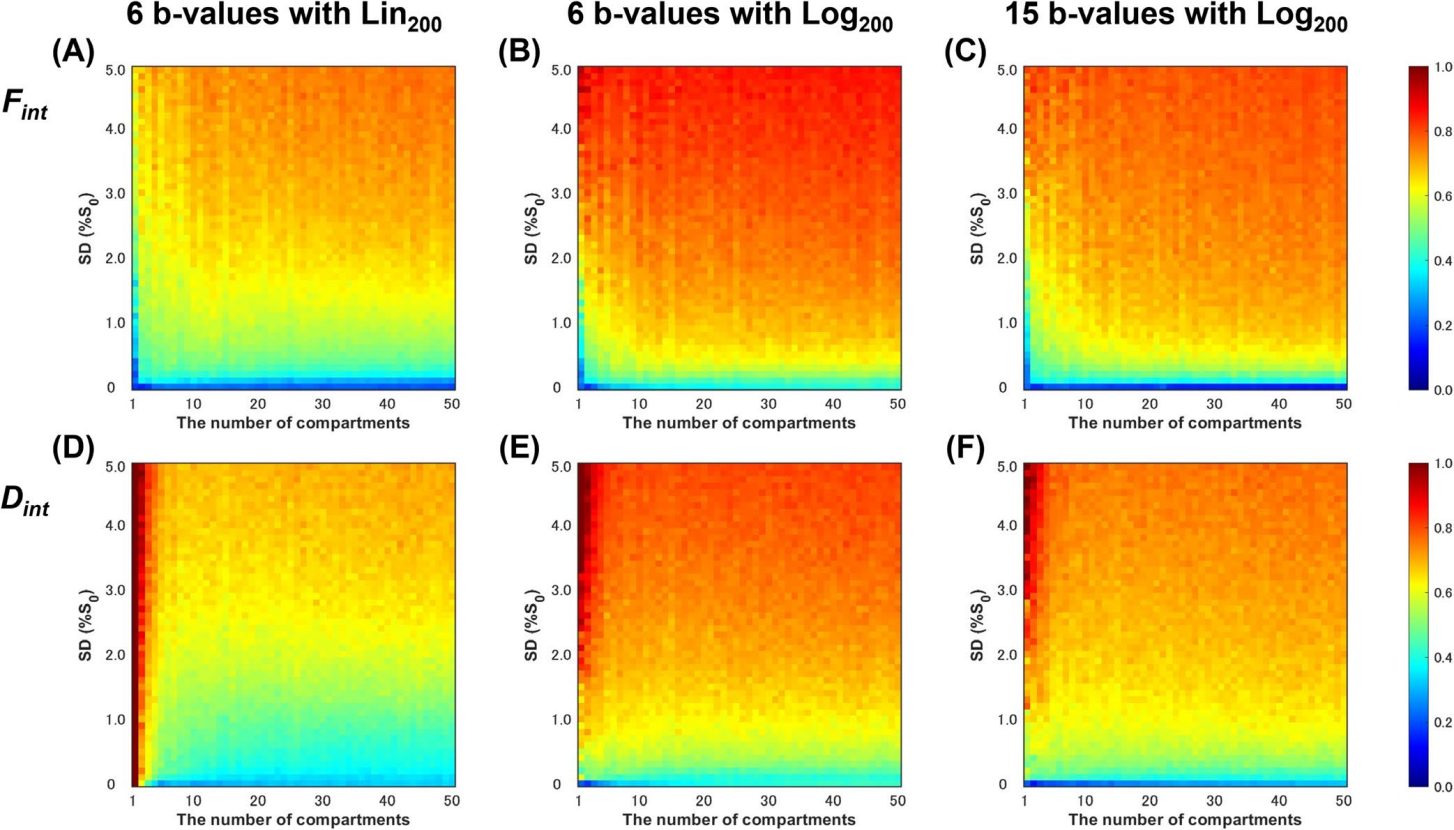
The normalized root mean squared error (nRMSE) between the ground truth and estimated values of *F*_int_ (upper) and *D*_int_ (lower). The left (A and D), middle (B and E), and right (C and F) columns display results from 6 *b*‐values with Lin_200_, 6 *b*‐values with Log_200_, and 15 *b*‐values with Log_200_, respectively.

Figure 3 presents the simulation results for *F*_int_ and *D*_int_. The overall trend of the nRMSE of *F*_int_ in response to increasing noise and the number of diffusion compartments remained consistent across all three methods (Figure 3A–C). The nRMSE increased with rising noise levels, particularly at higher numbers of diffusion compartments. Among the three methods, the highest accuracy was observed with six *b*‐values using Lin_200_. Similarly, the nRMSE pattern for *D*_int_ mirrored that of *F*_int_ (Figure 3D–F). However, all methods exhibited substantially elevated nRMSE values with a low number of diffusion compartments, especially with six *b*‐values using Lin_200_. Nevertheless, this setting demonstrated the highest accuracy based on the overall nRMSE behavior of *D*_int_ across the three methods. Therefore, Lin_200_ was selected for the in vivo study.

### 3.2 In Vivo Study

In iNPH, the CSO, FWM, and corona radiata each contained 68 ROIs (34 patients × 2). Among these, 33, 22, and 18 ROIs in the CSO, FWM, and corona radiata, respectively, were categorized as the iNPH_PVH_ group.

**FIGURE 4:**
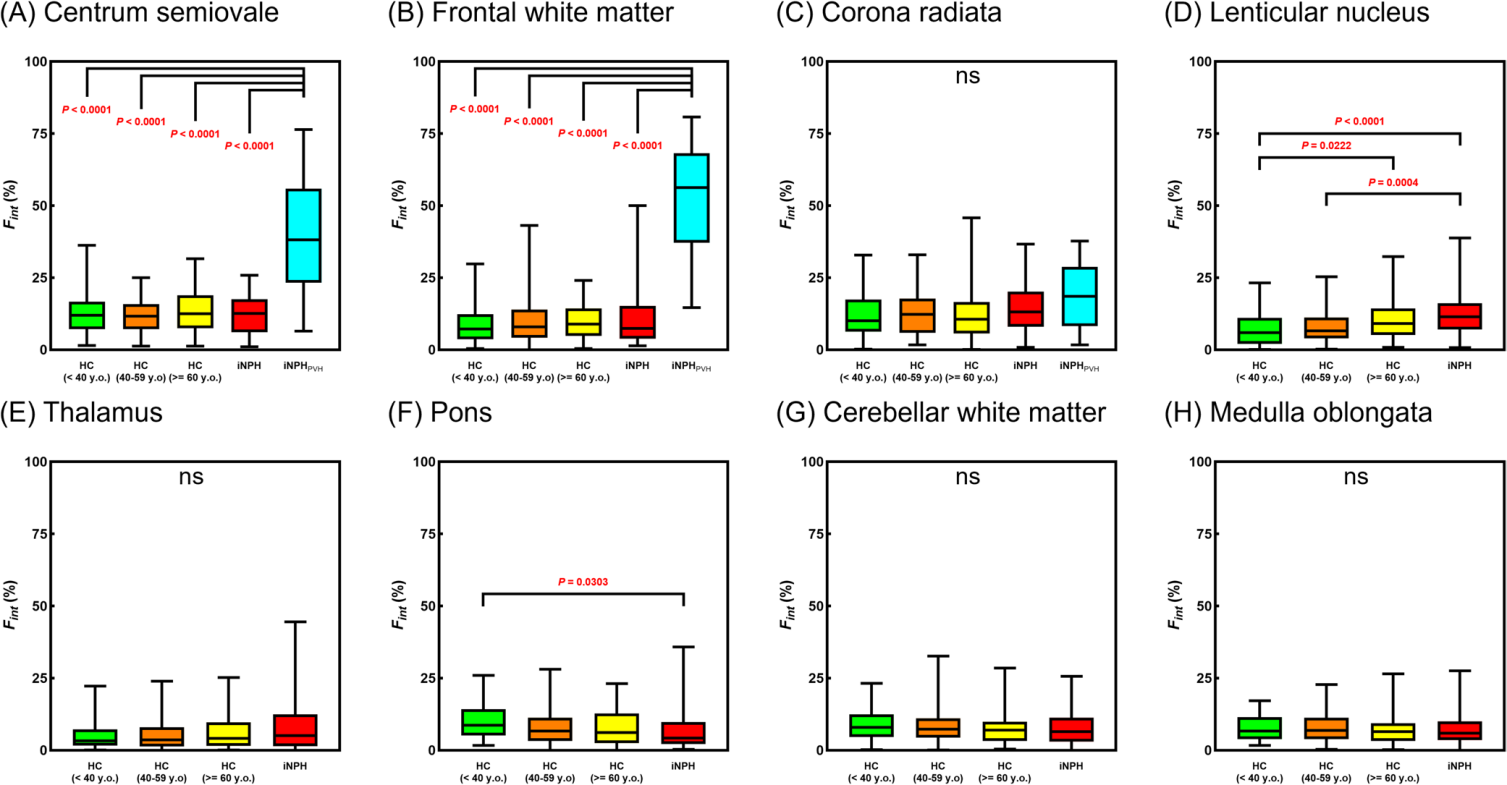
Group comparisons of *F*_int_ values measured in the centrum semiovale (A), frontal white matter (B), corona radiata (C), lenticular nucleus (D), thalamus (E), pons (F), cerebellar white matter (G), and medulla oblongata (H). Only *P*‐values indicating statistical significance are shown. ns, not significant; HC, healthy control; iNPH, idiopathic normal pressure hydrocephalus; PVH, periventricular hyperintensity.

**FIGURE 5:**
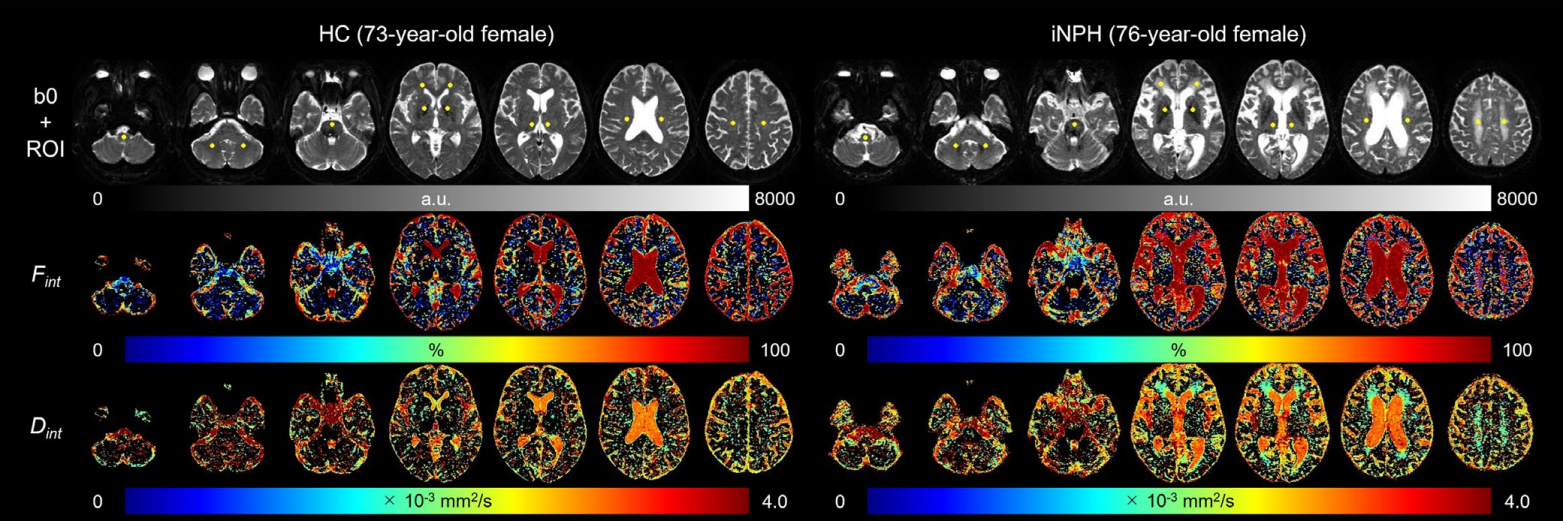
Representative b0 images with the locations of regions‐of‐interest (ROIs; first row), *F*_int_ maps (second row), and *D*_int_ maps (third row) obtained from a 73‐year‐old female healthy control (HC; left column) and a 76‐year‐old female patient with idiopathic normal pressure hydrocephalus (iNPH; right column).

Figure 4 and Tables 1 and 2 present the group comparison results for *F*_int_. In the CSO and FWM, the *F*_int_ values in the iNPH_PVH_ group (CSO, 40.3% ± 18.4% and FWM, 53.0% ± 18.9%) were significantly higher than those in the other groups. In the LN, *F*_int_ values in the iNPH group (13.1% ± 8.7%) were significantly greater than those in the HC group under 40 years (7.3% ± 5.8%) and those aged 40–59 years (8.1% ± 5.6%). In the pons, *F*_int_ values in the iNPH group (7.3% ± 7.8%) were significantly lower than those in the HC group under 40 years (10.4% ± 6.5%). Increases in *F*_int_ were clearly observed in PVH regions in iNPH (Figure 5).

**TABLE 1:** *F*_int_ and *D*_int_ values measured in the centrum semiovale, frontal white matter, corona radiata, lenticular nucleus, thalamus, pons, cerebellar white matter, and medulla oblongata.

	HC (< 40 years)	HC (40–59 years)	HC (≥ 60 years)	iNPH	iNPH_PVH_
*F*_int_ [%]					
Centrum semiovale	12.7 ± 7.0	11.6 ± 5.6	13.5 ± 7.5	12.6 ± 7.1	40.3 ± 18.4
Frontal white matter	8.6 ± 6.0	9.5 ± 7.4	9.7 ± 6.1	11.1 ± 9.9	53.0 ± 18.9
Corona radiata	11.7 ± 7.0	12.4 ± 7.4	12.7 ± 9.3	14.3 ± 8.4	19.7 ± 11.7
Lenticular nucleus	7.3 ± 5.8	8.1 ± 5.6	10.3 ± 7.0	13.1 ± 8.7	N/A
Thalamus	5.0 ± 4.9	5.2 ± 4.9	6.1 ± 6.0	8.6 ± 9.7	N/A
Pons	10.4 ± 6.5	8.0 ± 6.5	7.5 ± 6.0	7.3 ± 7.8	N/A
Cerebellar white matter	8.9 ± 5.6	8.5 ± 5.9	8.2 ± 6.4	8.2 ± 6.5	N/A
Medulla oblongata	7.8 ± 4.4	7.9 ± 5.4	7.1 ± 5.5	7.6 ± 5.7	N/A
*D*_int_ [× 10^−3^ mm^2^/s]					
Centrum semiovale	1.05 ± 0.45	1.02 ± 0.46	1.21 ± 0.49	0.97 ± 0.48	1.53 ± 0.63
Frontal white matter	0.85 ± 0.48	0.97 ± 0.56	0.97 ± 0.55	0.89 ± 0.56	1.64 ± 0.50
Corona radiata	0.92 ± 0.47	1.01 ± 0.41	1.10 ± 0.66	0.95 ± 0.45	1.17 ± 0.85
Lenticular nucleus	0.72 ± 0.47	0.75 ± 0.49	0.83 ± 0.47	0.90 ± 0.53	N/A
Thalamus	0.60 ± 0.43	0.68 ± 0.44	0.73 ± 0.53	0.76 ± 0.62	N/A
Pons	0.90 ± 0.49	0.77 ± 0.49	0.90 ± 0.61	0.73 ± 0.59	N/A
Cerebellar white matter	0.99 ± 0.50	0.91 ± 0.51	0.92 ± 0.49	0.87 ± 0.50	N/A
Medulla oblongata	0.86 ± 0.47	0.90 ± 0.53	0.76 ± 0.39	0.91 ± 0.42	N/A

*Note*: Mean ± Standard deviation. HC, healthy control; iNPH, idiopathic normal pressure hydrocephalus; PVH, periventricular hyperintensity.

**TABLE 2:** *P*‐values for the group comparison of *F*_int_ and *D*_int_ in the centrum semiovale (CSO), frontal white matter (FWM), corona radiata (CR), lenticular nucleus (LN), thalamus, pons, cerebellar white matter (Cbll WM), and medulla oblongata (MO).

		CSO	FWM	CR	LN	Thalumus	Pons	Cbll WM	MO
*F*_int_									
HC, < 40 years	HC, 40–59 years	> 0.9999	> 0.9999	> 0.9999	> 0.9999	> 0.9999	0.2862	> 0.9999	> 0.9999
HC, < 40 years	HC, ≥ 60 years	> 0.9999	> 0.9999	> 0.9999	0.0222	> 0.9999	0.2067	> 0.9999	> 0.9999
HC, < 40 years	iNPH	> 0.9999	> 0.9999	0.9564	< 0.0001	0.5083	0.0303	> 0.9999	> 0.9999
HC, < 40 years	iNPH_PVH_	< 0.0001	< 0.0001	0.0732	N/A	N/A	N/A	N/A	N/A
HC, 40–59 years	HC, ≥ 60 years	> 0.9999	> 0.9999	> 0.9999	0.3344	> 0.9999	> 0.9999	> 0.9999	> 0.9999
HC, 40–59 years	iNPH	> 0.9999	> 0.9999	> 0.9999	0.0004	0.8779	> 0.9999	> 0.9999	> 0.9999
HC, 40–59 years	iNPH_PVH_	< 0.0001	< 0.0001	0.1550	N/A	N/A	N/A	N/A	N/A
HC, ≥ 60 years	iNPH	> 0.9999	> 0.9999	> 0.9999	0.3194	> 0.9999	> 0.9999	> 0.9999	> 0.9999
HC, ≥ 60 years	iNPH_PVH_	< 0.0001	< 0.0001	0.1343	N/A	N/A	N/A	N/A	N/A
iNPH	iNPH_PVH_	< 0.0001	< 0.0001	> 0.9999	N/A	N/A	N/A	N/A	N/A
*D*_int_									
HC, < 40 years	HC, 40–59 years	> 0.9999	> 0.9999	0.4264	> 0.9999	0.8076	> 0.9999	> 0.9999	> 0.9999
HC, < 40 years	HC, ≥ 60 years	0.3806	> 0.9999	0.8372	0.6139	0.5638	> 0.9999	> 0.9999	> 0.9999
HC, < 40 years	iNPH	> 0.9999	> 0.9999	> 0.9999	0.1166	> 0.9999	0.3252	0.7152	> 0.9999
HC, < 40 years	iNPH_PVH_	0.0008	< 0.0001	> 0.9999	N/A	N/A	N/A	N/A	N/A
HC, 40–59 years	HC, ≥ 60 years	0.2876	> 0.9999	> 0.9999	> 0.9999	> 0.9999	> 0.9999	> 0.9999	> 0.9999
HC, 40–59 years	iNPH	> 0.9999	> 0.9999	> 0.9999	0.2491	> 0.9999	> 0.9999	> 0.9999	> 0.9999
HC, 40–59 years	iNPH_PVH_	0.0005	< 0.0001	> 0.9999	N/A	N/A	N/A	N/A	N/A
HC, ≥ 60 years	iNPH	0.2169	> 0.9999	> 0.9999	> 0.9999	> 0.9999	0.9435	> 0.9999	0.6835
HC, ≥ 60 years	iNPH_PVH_	0.2455	< 0.0001	> 0.9999	N/A	N/A	N/A	N/A	N/A
iNPH	iNPH_PVH_	0.0010	< 0.0001	> 0.9999	N/A	N/A	N/A	N/A	N/A

Abbreviations: HC, healthy control; iNPH, idiopathic normal pressure hydrocephalus; N/A, not available; PVH, periventricular hyperintensity.

**FIGURE 6:**
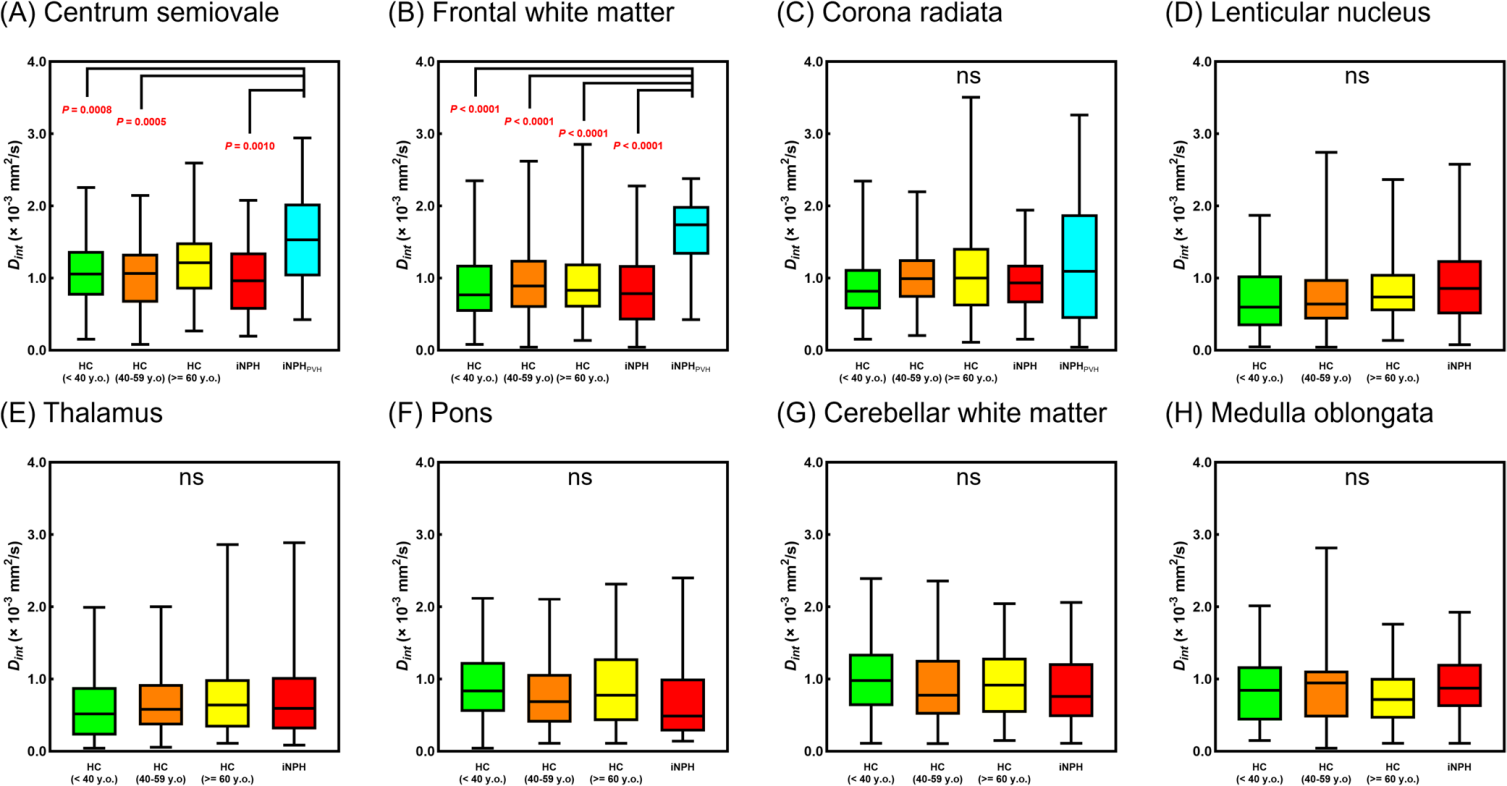
Group comparisons of *D*_int_ values measured in the centrum semiovale (A), frontal white matter (B), corona radiata (C), lenticular nucleus (D), thalamus (E), pons (F), cerebellar white matter (G), and medulla oblongata (H). Only *P*‐values indicating statistical significance are shown. ns, not significant; HC, healthy control; iNPH, idiopathic normal pressure hydrocephalus; PVH, periventricular hyperintensity.

Figure 6 and Tables 1 and 2 display the group comparison results for *D*_int_. In the CSO, the *D*_int_ values in the iNPH_PVH_ group (1.53 ± 0.63 [× 10^−3^ mm^2^/s]) were significantly higher than those in the HC group under 40 years (1.05 ± 0.45 [× 10^−3^ mm^2^/s]), those aged 40–59 years, and the iNPH group (1.02 ± 0.46 [× 10^−3^ mm^2^/s]). In the FWM, the *D*_int_ values in the iNPH_PVH_ group (1.64 ± 0.50 [× 10^−3^ mm^2^/s]) were significantly elevated compared with those in the other groups. However, no significant differences were observed in *D*_int_ values in other regions. Increased *D*_int_ was clear in PVH regions in iNPH (Figure 5).

**FIGURE 7:**
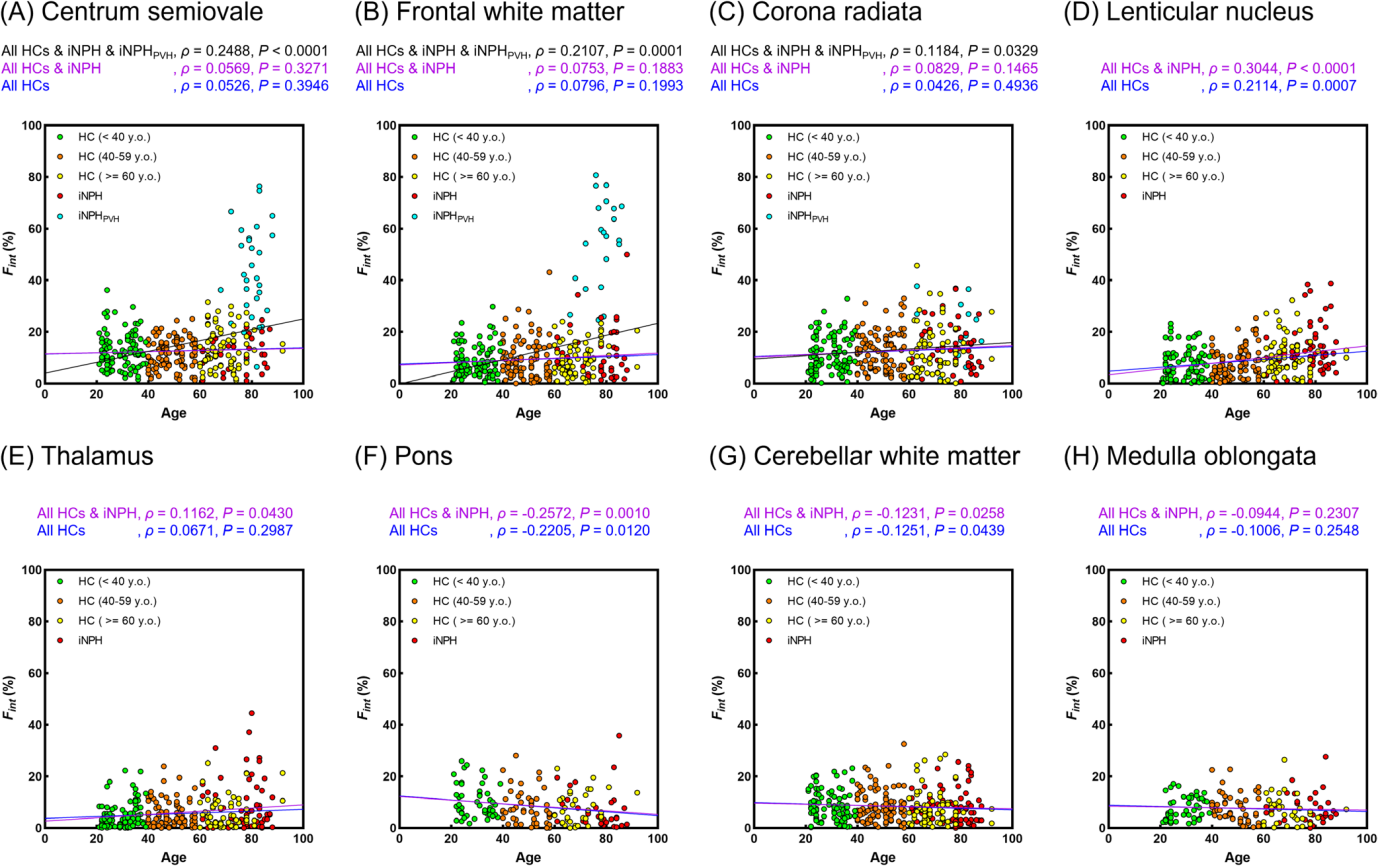
Correlation between *F*_int_ values and age in the centrum semiovale (A), frontal white matter (B), corona radiata (C), lenticular nucleus (D), thalamus (E), pons (F), cerebellar white matter (G), and medulla oblongata (H). The blue, purple, and black lines represent regression lines for all healthy controls (HCs), for all HCs and patients with idiopathic normal pressure hydrocephalus (iNPH), and for all data, including HCs, iNPH, and iNPH with periventricular hyperintensity (iNPH_PVH_), respectively. *ρ* represents the Spearman correlation coefficient.

**FIGURE 8:**
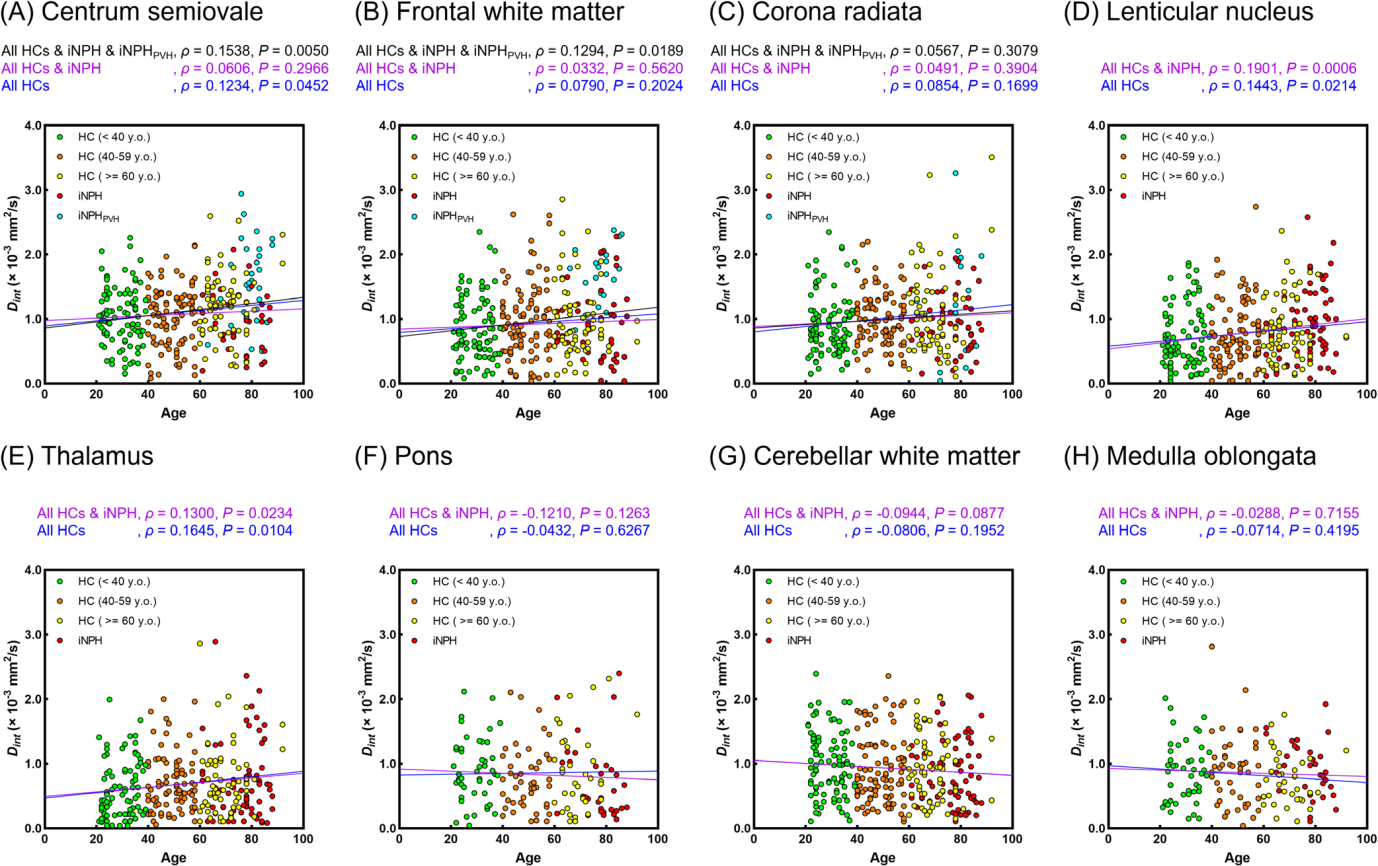
Correlation between *D*_int_ values and age in the centrum semiovale (A), frontal white matter (B), corona radiata (C), lenticular nucleus (D), thalamus (E), pons (F), cerebellar white matter (G), and medulla oblongata (H). The blue, purple, and black lines represent regression lines for all healthy controls (HCs), for all HCs and patients with idiopathic normal pressure hydrocephalus (iNPH), and for all data, including HCs, iNPH, and iNPH with periventricular hyperintensity (iNPH_PVH_), respectively. *ρ* represents the Spearman correlation coefficient.

Figures 7 and 8 illustrate the relationships between age and *F*_int_ and *D*_int_ values, respectively. In the LN, *F*_int_ values demonstrated a positive correlation with age, regardless of the iNPH group's inclusion (All HCs, *ρ* = 0.2114, All HCs and iNPH, *ρ* = 0.3044). Conversely, in the pons, *F*_int_ values exhibited a negative correlation with age, with regression lines remaining unchanged whether the iNPH group was included (All HCs, *ρ* = −0.2205, All HCs and iNPH, *ρ* = −0.2572). In the CSO, FWM, and corona radiata, significant correlations between *F*_int_ values and age were no longer observed when the iNPH_PVH_ group was excluded (CSO: All HCs, *p* = 0.3946, All HCs and iNPH, *p* = 0.3271; FWM: All HCs, *p* = 0.1993, All HCs and iNPH, *p* = 0.1883; and corona radiata: All HCs, *p* = 0.4936, All HCs and iNPH, *p* = 0.1465). Additionally, weak positive correlations between *D*_int_ values and age were identified in the LN (All HCs, *ρ* = 0.1443, All HCs and iNPH, *ρ* = 0.1901) and thalamus (All HCs, *ρ* = 0.1645, All HCs and iNPH, *ρ* = 0.1300).

## 4 Discussion

This study examined differences in ISF volume and diffusivity between iNPH and HCs and explored their correlation with age using SDA. A simulation study assessed the impact of *b*‐value and dictionary settings on the accuracy of *F*_int_ and *D*_int_, proposing an original dictionary setting to maintain accuracy, even with a limited number of non‐optimized *b*‐values. In the in vivo study, ISF volume and diffusivity were significantly increased in iNPH compared with HCs, specifically in the PVH regions within the CSO and FWM, whereas no significant correlations with age were identified in most measurement regions. These findings enhance the understanding of ISF dynamics in iNPH.

### 4.1 Simulation Study

In the simulation, numerous patterns of diffusion signal decay, including a wide range of noise levels, were analyzed. This process could lead to overfitting; however, NNLS is inherently robust against it, potentially reducing sensitivity to noise [31, 32].

The simulation results demonstrated that Lin_200_ achieved greater accuracy than Log_200_ in estimating both *F*_int_ and *D*_int_, even with a limited number of *b*‐values. This superior performance is attributed to variations in the distribution and number of DCs across the diffusion components within the dictionary. Specifically, Log_200_ contained 59, 21, and 120 DCs for the parenchymal diffusion, intermediate diffusion, and microvascular pseudo‐diffusion components, respectively, whereas Lin_200_ included 66, 68, and 66 DCs. Therefore, Lin_200_ had approximately three times the number of DCs within the intermediate diffusion component range compared with Log_200_, thereby improving the accuracy of *F*_int_ and *D*_int_ estimation. Nevertheless, determining an optimal dictionary configuration applicable across various pathological conditions remains challenging due to the unknown physiological and pathological distribution of DCs in the brain. Consequently, this study assumed a uniform distribution for the ground truth DCs, which may have introduced a bias favoring Lin_200_. Despite this limitation, the findings indicate that customizing the dictionary for the target diffusion component may enhance estimation accuracy.

The large nRMSE of *D*_int_ observed with a limited number of diffusion compartments was attributed to the spectral behavior near the boundary values of the intermediate diffusion component (1.5 and 4.0 [× 10^−3^ mm^2^/s]), particularly pronounced near the upper limit. The spacing between DCs in the dictionary near this boundary (e.g., Log_200_, *D*_80_ = 3.872 and *D*_81_ = 4.055; Lin_200_, *D*_134_ = 4.000 and *D*_135_ = 19.091 [× 10^−3^ mm^2^/s]) may contribute to this issue. SDA represents diffusion signal decay as the optimal combination of DCs within a dictionary. In this context, Log_200_ provides a better fit than Lin_200_ for spectra that slightly exceed the upper limit because the difference between the last DC in the intermediate diffusion component and the first DC in the microvascular pseudo‐diffusion component is smaller in Log_200_ (i.e., *D*_80_ and *D*_81_) than in Lin_200_ (i.e., *D*_134_ and *D*_135_). However, as the number of compartments increases, this effect diminishes due to the reduced influence of DCs near the boundary values on the overall diffusion signal decay. For both Lin_200_ and Log_200_, accuracy declined with increasing noise levels due to the predominant impact of noise‐induced signal fluctuations. Given that a voxel where diffusion signal decay is described by a single DC is rare, the overall trend in nRMSE suggests that Lin_200_ achieved greater accuracy than Log_200_ in estimating *D*_int_.

### 4.2 In Vivo Study

This study included three HCs with incidentally discovered small unruptured intracranial aneurysms. Although the potential impact of these aneurysms on ISF dynamics was considered, no studies currently demonstrate such an effect. Therefore, regarding small unruptured aneurysms as unlikely to affect ISF dynamics has limitations. Furthermore, potential confounding factors, such as vascular risk factors, medication effects, or other neurodegenerative conditions, were not evaluated. Future studies should assess the impact of these confounding factors on ISF dynamics.

In this study, the ROI template was created for semi‐automatic measurements. However, distortion correction was not possible due to the unavailability of b0 images with opposing phase encoding directions, given the retrospective nature of this study. To address this, slight manual adjustments were made to the ROI template, which may have introduced measurement bias. Furthermore, fixed‐size ROIs may impose partial volume effects, further contributing to bias. A volume‐of‐interest‐based approach using lesion segmentation may offer a more accurate evaluation of ISF volume and diffusivity [33].

In the CSO and FWM, *F*_int_ values in the iNPH_PVH_ group were significantly higher than in the other groups, with no significant correlations between age and *F*_int_ values. These findings align with previous observations, indicating PVH increases ISF volume [19, 34]. This reflects a pathological condition of iNPH, where CSF permeates brain tissue without passing through the PVS due to ependymal lining disruption on the ventricular surface, which is caused by mechanical stretching from ventriculomegaly [35, 36, 37, 38]. Additionally, increased *F*_int_ is associated with enlarged PVS (ePVS) because *F*_int_ represents the fraction of components with relatively large DCs within a voxel. This complicates the differentiation between ISF volume increases and ePVS, as both are assumed to have relatively large DCs. Consequently, the effect of ePVS may counteract the effect of PVH when comparing *F*_int_ values of iNPH and iNPH_PVH_ groups to the HC group aged ≥ 60 years. This is because ePVS in the CSO of iNPH is less likely to occur than in age‐matched controls due to mechanical compression from upward brain displacement [39, 40]. However, in the CSO, no significant difference in *F*_int_ values was found between the iNPH and HC groups aged ≥ 60 years, suggesting that the effect of PVH predominates in the significant increase in *F*_int_ values of the iNPH_PVH_ group. Similarly, *D*_int_ values were significantly larger in the iNPH_PVH_ group and showed no correlation with age. The increase in *D*_int_ values reflects enhanced water diffusivity due to increased ISF volume.

In the corona radiata, although *F*_int_ and *D*_int_ values showed no significant differences, they tended to be higher in the iNPH_PVH_ group compared with the others, with no significant correlations with age. While the mechanisms underlying these results are considered to be similar to those in the CSO and FWM, the lack of significance remains unclear. One possible explanation is that PVH results from the thinning of the ependymal lining on the ventricular surface due to mechanical stretching [35, 36]. Additionally, the lateral ventricles in iNPH expand cranially, and the frontal horns of the lateral ventricles are a known predilection site for PVH [37, 41]. These observations suggest that the corona radiata in iNPH is less susceptible to CSF permeation into brain tissue than the CSO and FWM. Furthermore, although contamination between WM hyperintensity and isointensity was minimized by slight modifications to the ROI template, PVH and deep WM hyperintensity were not differentiated. This may have contributed to the lack of significant differences in *F*_int_ and *D*_int_ values in the iNPH_PVH_ group despite a report indicating no clear evidence for distinguishing WM hyperintensity subtypes [42].

In the LN, *F*_int_ values were significantly increased in the iNPH group and positively correlated with age. These results may reflect age‐related ePVS rather than changes in ISF dynamics, as *F*_int_ represents the fraction of components with relatively large DCs within a voxel and cannot differentiate between increases in ISF volume and ePVS [43, 44]. Furthermore, while *D*_int_ did not significantly increase in the iNPH group, it was significantly correlated with age. This may also indicate age‐related ePVS, and the lack of significant differences in *D*_int_ values could be attributed to a less pronounced increase in water volume within the voxels compared with that observed in the PVH.

In the pons, although *F*_int_ values showed a weak negative correlation with age, the reason for this remains unclear because an increase in ISF volume is typically expected with aging or pathological conditions [45]. Additionally, *F*_int_ values in the iNPH group were significantly lower than those in the HC group under 40 years and tended to be smaller than those in the other HC groups. This suggests reduced ISF volume in the pons of the iNPH group, potentially contradicting a previous study that reported delayed tracer clearance in the brainstem, including the pons, in iNPH, where increased ISF volume is expected [45, 46]. Therefore, further studies using alternative techniques are needed to verify age‐related changes in ISF dynamics in this region [47].

Although age‐related impairment of ISF dynamics may contribute to the pathogenesis of iNPH, this study suggests three possibilities: (1) *F*_int_ and *D*_int_ simply represent ISF volume and diffusivity, respectively, and do not necessarily reflect differences in ISF dynamics between iNPH and HCs; (2) ISF dynamics in iNPH may not differ significantly from those in HCs; and (3) while ISF dynamics in iNPH are indeed altered and *F*_int_ and *D*_int_ can reflect its pathological changes, these changes may be subtle and undetectable by SDA [10, 47, 48, 49]. Further research is needed to determine whether the findings and hypotheses of this study are valid or if the issue lies in the detection capability of SDA regarding changes in ISF dynamics. If the findings are valid, *F*_int_ and *D*_int_ could detect glymphatic dysfunction early, thereby enabling timely therapeutic intervention, monitoring of disease progression, and predicting therapeutic outcomes.

### 4.3 Limitations

First, this study used a single‐center, single‐vendor, single‐scanner, single‐field‐strength dataset with a small sample size. Further studies are necessary to validate the results of this study in comparison to other imaging techniques. Second, the distributions of gender and age in the HC group aged ≥ 60 years did not match those in the iNPH group, with the mean age in the iNPH group being significantly higher [7]. Third, while b0 images were used for ROI categorization, fluid‐attenuated inversion recovery or spin‐echo‐based T_2_‐weighted images would be preferable. Finally, future studies should assess reproducibility, repeatability, scanner variability, and inter‐rater reliability.

### 4.4 Conclusion

Spectral diffusion analysis provides valuable insights into ISF dynamics in iNPH. Changes in ISF volume and diffusivity were observed in the PVH of the CSO and FWM due to CSF permeation through disrupted ependymal cells on the ventricular surface into brain tissue. However, ISF dynamics in iNPH may not differ significantly from those in HCs outside the PVH. Age‐related changes in ISF volume and diffusivity were rarely observed, except in the ePVS of the LN.

## Supporting information


**Data S1.** Supporting Information.
