# Supplementary material for: Evaluation of Interstitial Fluid Volume and Diffusivity in Patients With Idiopathic Normal Pressure Hydrocephalus Using Spectral Diffusion Analysis
Source: J Magn Reson Imaging. 2025 Jul 4;62(4):1092–104. doi: 10.1002/jmri.29834 (PMC12435118; doi:10.1002/jmri.29834)
Supplement: Supplementary file 1 — Data S1. Supporting Information. [file JMRI-62-1092-s001.docx]

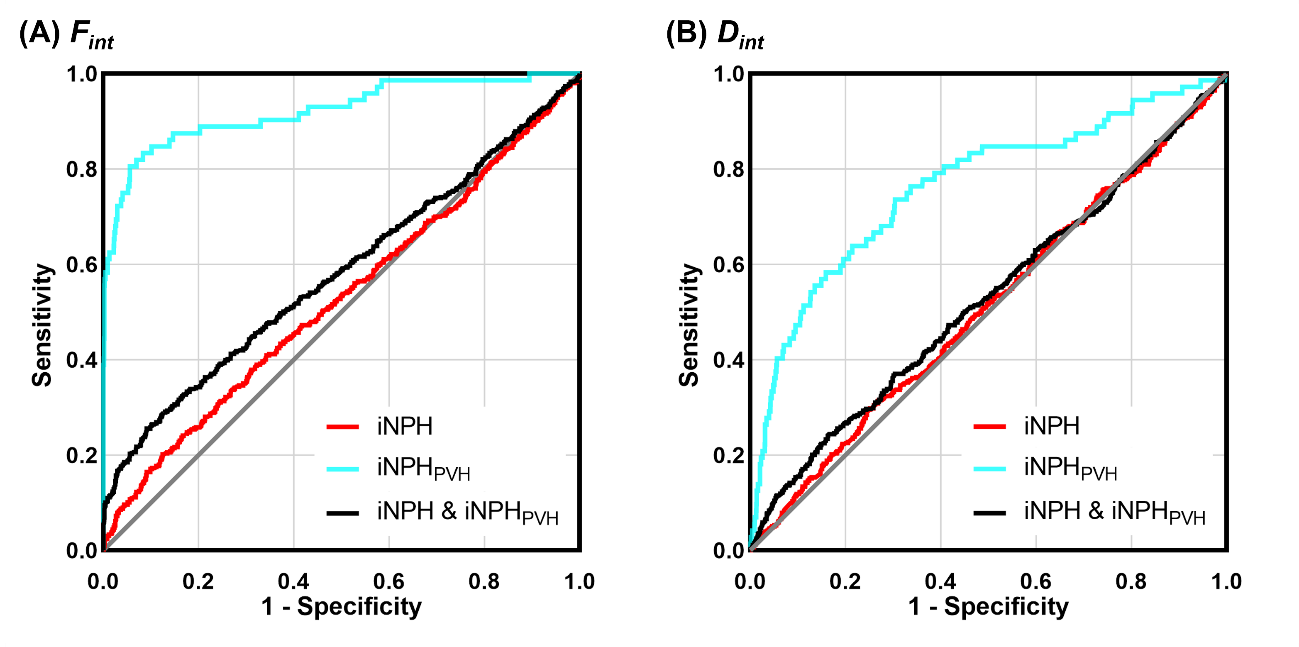


**Supporting Figure 1.** Receiver operating characteristic curves for (A) *F_int_* and (B) *D_int_*. iNPH, idiopathic normal pressure hydrocephalus; iNPH_PVH_, idiopathic normal pressure hydrocephalus periventricular hyperintensity

**Supporting Table 1.** Results of receiver operating characteristic analysis

|  | AUC  [95% CI] | Cutoff value  (Youden index) | Sensitivity  [95% CI] | Specificity  [95% CI] | *P*-value |
| --- | --- | --- | --- | --- | --- |
| *F_int_* [%] |  |  |  |  |  |
| iNPH | 0.5274  [0.4965–0.5583] | 17.4 | 20.1  [16.7–24.1] | 87.5  [86.0–88.9] | 0.0665 |
| iNPH_PVH_ | 0.9225  [0.8820–0.9630] | 19.8 | 83.3  [73.1–90.2] | 91.7  [90.4–92.8] | < 0.0001 |
| iNPH & iNPH_PVH_ | 0.5812  [0.5515–0.6109] | 17.4 | 28.9  [25.2–32.9] | 87.5  [86.0–88.9] | < 0.0001 |
| *D_int_* [× 10^-3^ mm^2^/s] |  |  |  |  |  |
| iNPH | 0.5094  [0.4795–0.5393] | 0.50 | 29.3  [25.3–33.7] | 75.4  [73.5–77.2] | 0.5305 |
| iNPH_PVH_ | 0.7630  [0.6989–0.8271] | 1.09 | 73.6  [62.4–82.4] | 69.7  [67.6–71.6] | < 0.0001 |
| iNPH & iNPH_PVH_ | 0.5277  [0.4989–0.5565] | 1.35 | 24.4  [20.9–28.2] | 83.5  [81.9–85.1] | 0.0491 |

AUC, area under the curve; CI, confidence intervals.
